# Fabrication of Highly Conductive Porous Fe_3_O_4_@RGO/PEDOT:PSS Composite Films via Acid Post-Treatment and Their Applications as Electrochemical Supercapacitor and Thermoelectric Material

**DOI:** 10.3390/polym15163453

**Published:** 2023-08-18

**Authors:** Luyao Gao, Fuwei Liu, Qinru Wei, Zhiwei Cai, Jiajia Duan, Fuqun Li, Huiying Li, Ruotong Lv, Mengke Wang, Jingxian Li, Letian Wang

**Affiliations:** 1 College of Physics and Electronic Engineering, Xinyang Normal University, Xinyang 464000, China; 2 Key Laboratory of Advanced Micro/Nano Functional Materials of Henan Province, Xinyang Normal University, Xinyang 464000, China; 3 Energy-Saving Building Materials Innovative Collaboration Center of Henan Province, Xinyang Normal University, Xinyang 464000, China

**Keywords:** PEDOT:PSS, Fe_3_O_4_, GO, composite film, supercapacitor, thermoelectric

## Abstract

As a remarkable multifunctional material, ferroferric oxide (Fe_3_O_4_) exhibits considerable potential for applications in many fields, such as energy storage and conversion technologies. However, the poor electronic and ionic conductivities of classical Fe_3_O_4_ restricts its application. To address this challenge, Fe_3_O_4_ nanoparticles are combined with graphene oxide (GO) via a typical hydrothermal method, followed by a conductive wrapping using poly(3,4-ethylenedioxythiophene):poly(styrene sulfonic sulfonate) (PEDOT:PSS) for the fabrication of composite films. Upon acid treatment, a highly conductive porous Fe_3_O_4_@RGO/PEDOT:PSS hybrid is successfully constructed, and each component exerts its action that effectively facilitates the electron transfer and subsequent performance improvement. Specifically, the Fe_3_O_4_@RGO/PEDOT:PSS porous film achieves a high specific capacitance of 244.7 F g^−1^ at a current of 1 A g^−1^. Furthermore, due to the facial fabrication of the highly conductive networks, the free-standing film exhibits potential advantages in flexible thermoelectric (TE) materials. Notably, such a hybrid film shows a high electric conductivity (σ) of 507.56 S cm^−1^, a three times greater value than the Fe_3_O_4_@RGO component, and achieves an optimized Seebeck coefficient (S) of 13.29 μV K^−1^ at room temperature. This work provides a novel route for the synthesis of Fe_3_O_4_@RGO/PEDOT:PSS multifunctional films that possess promising applications in energy storage and conversion.

## 1. Introduction

Considering the rapid growth of the world’s economy and the continual consumption of fossil fuels, green energy (such as wind, hydropower, thermoelectric, etc.) and electrical energy storage devices are in urgent need for many applications such as portable electronic devices and electric vehicles [1,2,3,4,5,6]. Among the variety of energy storage devices, supercapacitors (SCs) have attracted widespread attention for their high power density, reliable safety, outstanding cycling stability, and low cost [2,7,8,9,10]. Generally, there are two kinds of supercapacitors: electrochemical double-layer capacitors (EDLCs) and pseudocapacitors. While EDLCs store electricity through the double-layer effect, the pseudocapacitor works through a fast redox reaction, which is essential for harvesting outstanding capacitive ability [11,12,13].

To explore desired electrode materials for supercapacitors, many efforts have been put into researching transition metal oxides, such as Fe_3_O_4_, Fe_2_O_3_, Co_3_O_4_, RuO_2_, MnO_2_, etc. [10,14,15,16,17,18]. Among them, iron ferrite of Fe_3_O_4_ has been proposed as a potential supercapacitor material because of its high specific capacitance, easy redox reaction, rich natural storage, and environmental friendliness [10]. Nevertheless, Fe_3_O_4_ has a low conductivity in nature, which limits its electrochemical performance. Furthermore, it remains a challenge to avoid nanoparticle agglomeration during the preparation of electrode materials. In order to solve the above problems, Fe_3_O_4_ is commonly combined with carbon-based materials, especially graphene and CNTs. For instance, through a layer-by-layer method, the obtained Fe_3_O_4_/RGO multilayer electrodes exhibited a specific capacitance of 151 F g^−1^ when a current density of 0.9 A g^−1^ was used, and after 1000 cycles, the capacitance retained 85% of its original value, indicating good cycling stability [19]. A sandwich-like Fe_3_O_4_/MnO_2_/RGO nanocomposite was explored and the value of specific capacitance reached 77.5 F g^−1^ at 0.5 A g^−1^ and kept 35 F g^−1^ at 20 A g^−1^ in 1 M Na_2_SO_4_ [20]. CNT/Fe_3_O_4_ nanocomposites synthesized through the hydrothermal method also achieved a specific capacitance of 117.2 F g^−1^ at 10 mA cm^−2^ in a 6 M KOH electrolyte [21]. A novel BRGO/Fe_3_O_4_-MWCNT hybrid nanocomposite was successfully fabricated and possessed good supercapacitance performance (165 F g^−1^ at a current density of 2 A g^−1^) [22]. Not only that, the obtained BRGO/Fe_3_O_4_-MWCNT composites also possessed a high photo degradation efficiency. As conductive frameworks, carbon materials in these strategies effectively avoid the collapse of the nano-Fe_3_O_4_ particles and thus improve their electrochemical properties. However, to realize large capacitance and practical applications requires high mass loading of active Fe_3_O_4_, which in turn increases the electrode resistance and thus limits the performance characteristics of the composite electrodes. In addition, binder materials like polytetrafluoroethylene (PTFE) and polyvinylidene fluoride (PVDF) are frequently used during the preparation of metal-oxide-based nanocomposite films for the preparation of flexible composite materials [23,24]. However, these binders are nonconductive and decrease the electrical conductivity of the electrodes.

To address these issues, one promising strategy is to incorporate Fe_3_O_4_ nanoparticles onto carbon-based frameworks and coat them with conducting polymers to form highly interconnected networks for charge transformation. Thus, there is an urgent demand for highly conductive binders that can further disperse the packed Fe_3_O_4_/carbon nanostructures. Among various conducting polymers, PEDOT:PSS is water soluble and can be used as a binder that is capable of dispersing carbon-based materials and/or other kinds of nanomaterials in water. Additionally, PEDOT:PSS can achieve high conductivities via incorporating additives (such as organic solvents [25,26], ionic liquids [27], and inorganic salts [28], etc.) and post-treatment through polar solvents (e.g., DMSO [29,30], EG [31,32], etc.) or acids [33,34]. It is believed that the Fe_3_O_4_/carbon/PEDOT:PSS composite can be employed as an excellent capacity electrode material that possesses considerable potential application in energy storage devices. Furthermore, such a unique structure can effectively increase the conductivity of the composite material, and its potential application in energy conversion technologies, such as thermoelectric, photoelectric, and thermal sensor, can also be expected.

Herein, we construct a ternary system based on Fe_3_O_4_@RGO/PEDOT:PSS; the graphene oxide (GO) in the compound acts as a base supporting material, while the PEDOT:PSS serves as a highly conductive wrapping material. Notably, after acid treatment, the as-prepared hybrid composite is easily stripped from the glass substrate and forms porous, highly conductive, and flexible electrode films. Benefiting from the large-scale construction of porous structures and the highly connected conducting networks, the Fe_3_O_4_@RGO/PEDOT:PSS electrodes exhibit a high specific capacitance of 244.7 F g^−1^ at 1 A g^−1^, and a good rate capability that remains 146.0 F g^−1^ at 10 A g^−1^. Except for energy storage, the constructed hybrid films can also be used as thermoelectric (TE) materials, which are capable of converting low-grade and/or waste heat into electricity, making them an important source of green energy. A dimensionless figure of merit, ZT = S^2^σT/κ, is usually applied to evaluate the TE materials’ conversion efficiency, in which σ is the electrical conductivity of the material, S stands for the Seebeck coefficient, and T and κ represent absolute temperature and thermal conductivity, respectively. For polymers and their composites, the thermal conductivity is relatively low (lower than that of the inorganic TE materials by almost one to three orders of magnitude). Therefore, the power factor S^2^σ is a good approximation for comparing organic and hybrid thermoelectric materials. The experiment results reveal that the Fe_3_O_4_@RGO/PEDOT:PSS hybrid films possess better TE properties than those of their single components. The related mechanism is also discussed in detail.

## 2. Materials and Methods

### 2.1. Materials

PEDOT:PSS (PH1000, Mw = 326.388) was purchased from Heraeus Company (Hanau, Germany); both ferroferric oxide nanoparticles (Fe_3_O_4_, Mw = 231.54 g/mol, around 20 nm particle size) and lithium sulfate (Li_2_SO_4_, Mw = 109.94 g/mol) from Beijing InnoChem Science & Technology Co., Ltd. (Beijing, China); GO aqueous solution (5 mg/mL) from Suzhou Tanfeng Graphene Technology Co., Ltd. (Suzhou, China); perchloric acid (HClO_4_, 70~72%, ~1.76 g/mL) and hydroiodic acid (HI, 57%, 5.23 g/mL) were obtained from Tianjin DaMao Chemical Reagent Factory (Tianjin, China) and Shanghai Mclean Biochemical Technology Co., Ltd. (Shanghai, China), respectively. Water used in this work was all deionized (DI) water (its resistance is around 18.2 MΩ cm). All the agents employed in the experiments were utilized directly.

### 2.2. Preparation of Fe_3_O_4_@GO and Fe_3_O_4_@RGO

The Fe_3_O_4_@GO composites were prepared according to a typical hydrothermal method. Firstly, Fe_3_O_4_ nanoparticles (12.5 mg) were added to 12.5 mL of GO aqueous solution (2 mg mL^−1^). Then, 12.5 mL of deionized water was added and the mixture was shaken well. After 30 min of sonication, the homogenous solution obtained was transferred to a 50 mL Teflon-lined steel autoclave and heated to 180 °C for 12 h. Subsequently, the steel autoclave was taken out and cooled to room temperature. The black sediment was collected and allowed to freeze-dry for 24 h. Finally, ~20.3 mg of black-brown fluffy product was obtained. For comparison, some Fe_3_O_4_@GO powder was also treated using HI for preparation of Fe_3_O_4_@RGO.

### 2.3. Preparation of Fe_3_O_4_@GO/PEDOT:PSS Composite Films

The Fe_3_O_4_@GO/PEDOT:PSS composite films were prepared via a drop-coating method; 25 mg of Fe_3_O_4_@GO lyophilized powder was dispersed in 10 mL PEDOT:PSS solution (1 mg mL^−1^). The obtained suspension was sonicated for about 1 h at room temperature. Thereafter, the as-prepared solution was allowed to drop onto a precleaned glass substrate, followed by a drying process at room temperature.

### 2.4. Preparation of Fe_3_O_4_@RGO/PEDOT:PSS Free-Standing Films

For fabrication of the free-standing Fe_3_O_4_@RGO/PEDOT:PSS films, an acid post-treatment was applied. The as-prepared Fe_3_O_4_@GO/PEDOT:PSS hybrid films were first immersed in HClO_4_ for 24 h. After that, the films were washed using DI water, followed by an air-drying process. To ensure good conductivities of the electrodes, the samples were further treated using HI with a similar procedure as described above. During immersion using HI, a chemical reductant, GO, can be effectively reduced to RGO. Finally, the free-standing Fe_3_O_4_@RGO/PEDOT:PSS hybrid films were successfully fabricated.

### 2.5. Characterization and Measurements

A Hitachi 4800 field emission scanning electron microscope (FE-SEM) was employed to analyze the morphologies of the films (Hitachi Limited, Tokyo, Japan). A Thermo K-Alpha X-ray photoelectron spectroscope (XPS) was used to determine the electron-binding energies of the samples (Thermo Fisher Scientific, Shanghai, China). Raman spectroscopy was performed using a LabRAM HR Evolution instrument with a 532 nm laser (HORIBA, Shanghai, China). An SDT Q600 (TA Instruments, New Castle, DE, USA) was applied for thermogravimetric analysis (TGA). An electrochemical performance analysis of the samples was conducted using a CHI 660E electrochemical workstation (Shanghai CH instruments Co., Shanghai, China). The thermoelectric properties were measured by employing a thin-film thermoelectric test system (MRS-3 M, Wuhan Joule Yacht Science &Technology Co., Ltd., Wuhan, China).

In this study, cyclic voltammogram (CV) curves, galvanostatic charge–discharge (GCD) curves, and electrochemical impedance spectroscopy (EIS) were collected via a three-electrode system in which a platinum mesh, saturated calomel electrode, and the as-prepared sample were used as the counter electrode, reference electrode, and working electrode, respectively. A 1 M Li_2_SO_4_ aqueous solution was used as the electrolyte. The specific capacitance (Cg, F g^−1^) of the samples was calculated with the formula Cg = IΔt/mΔV, where I stands for constant discharge current (A), Δt for discharge time (s), and m and ΔV for the mass of the film sample and the potential window, respectively.

## 3. Results and Discussion

### 3.1. Fabrication of Fe_3_O_4_@RGO/PEDOT:PSS Free-Standing Film

**Figure 1:**
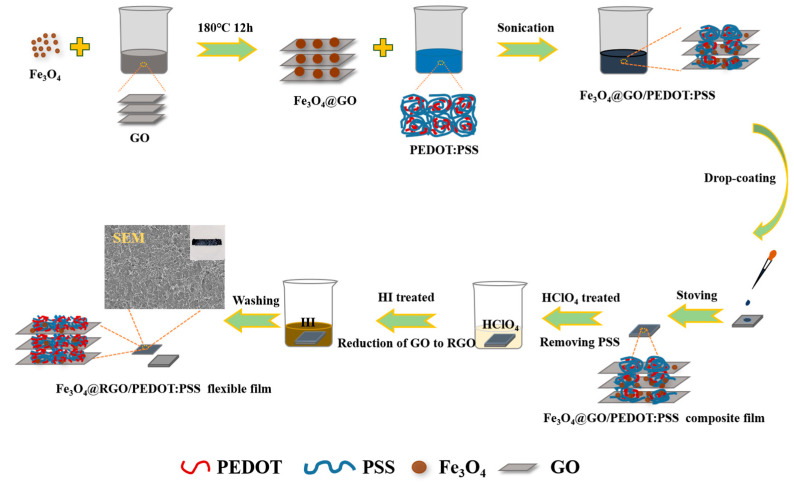
Schematic illustration of the fabrication process of the free-standing Fe_3_O_4_@RGO/PEDOT:PSS film.

Figure 1 provides a schematic illustration of how Fe_3_O_4_@RGO/PEDOT:PSS free-standing films are fabricated. First, Fe_3_O_4_ nanoparticles are combined with GO nanosheets for preparation of the Fe_3_O_4_@GO nanocomposites. After washing and freeze-drying, the Fe_3_O_4_@GO framework is successfully fabricated. For further improvement of the electric conductivity, a PEDOT:PSS conducting polymer is incorporated for conductive wrapping. With a subsequent HClO_4_ treatment, the conductivity of the PEDOT:PSS is dramatically improved, owing to the removal of nonconductive PSS and the formation of ordered molecular packing. Furthermore, a secondary acid treatment is conducted using HI, an efficient reducing agent that can eliminate the attached oxygen-containing functional groups and enhance the electrical conductivity of GO. Moreover, the Fe_3_O_4_ component is partly etched during the acid treatment, which contributes to the construction of interconnected porous nanostructures. Based on the above analysis, we infer that the obtained flexible, free-standing, and porous film should possess good electrochemical properties, which will be discussed latterly.

### 3.2. Structure Characterization and Analysis

**Figure 2:**
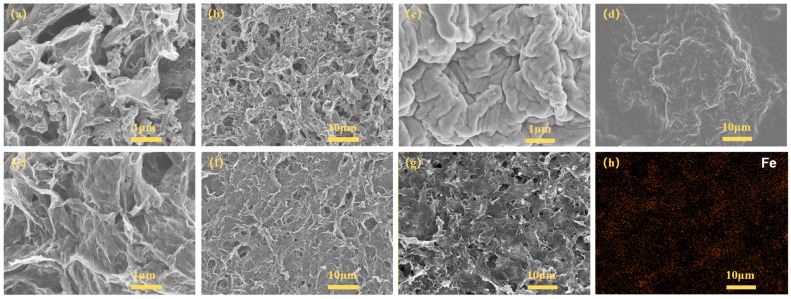
SEM images of (**a**,**b**) the Fe_3_O_4_@GO composites; (**c**,**d**) Fe_3_O_4_@GO/PEDOT:PSS hybrid films as prepared; (**e**–**g**) Fe_3_O_4_@RGO/PEDOT:PSS flexible free-standing films after acid treatment; (**h**) the corresponding EDS mapping image of (**g**) Fe_3_O_4_@RGO/PEDOT:PSS.

First, SEM was applied to examine the surface morphology of the obtained precursors and the final products. The as-prepared Fe_3_O_4_@GO exhibits an interconnected, highly porous microstructure, as shown in Figure 2a,b. First, the ultrasonic process results in homogeneous dispersion of the GO aqueous solution and Fe_3_O_4_ nanoparticles. And the subsequent liquid interfacial polymerization, under high pressure and temperature, allows the Fe_3_O_4_ structures to grow uniformly on the surface of the GO nanosheets. Finally, the freeze-drying procedure maintains the lamellar structure of the GO, and a homogeneous porous nanostructure is successfully constructed. With the introduction of the conductive PEDOT:PSS polymer, the gained composite films no longer possess a porous architecture. As is well known, PEDOT:PSS is water soluble and can wrap around the surface of the Fe_3_O_4_@GO precursor, forming an electrically conductive polymer shell. As depicted in Figure 2c,d, it can be observed visually that the Fe_3_O_4_@GO/PEDOT:PSS film shows a highly crumpled surface, which should be derived from the Fe_3_O_4_@GO core and PEDOT:PSS shell.

Notably, the superficial structure of the hybrid has changed significantly after the acid treatment. As exhibited in Figure 2e,f, the porous nanostructure is rediscovered in the complex architecture of Fe_3_O_4_@RGO/PEDOT:PSS, which can be ascribed to the acid treatment. As those meso-/macropores would provide a large surface area, electrolyte transport and access to active sites could be enhanced during the charging/discharging process. EDS element mapping was also conducted, and the results (Figure 2g,h) clearly reveal that the Fe element is evenly distributed in the Fe_3_O_4_@RGO/PEDOT:PSS sample. All these features would be beneficial for enhancing the supercapacitive performance of the hybrid films. On the other hand, when it is used as a thermoelectric material, the porous nanostructure is of importance in suppressing the thermal conductivity, whereas the large number of holes is not conducive to the formation of conductive networks and thus leads to a reduction in electrical conductivity and a decrease in mechanical properties. And thus, the presence of a conductive binder becomes very important. Furthermore, the introduction of conductive polymers is conducive to achieving relatively low thermal conductivity (≤1 W·m^−1^·K^−1^ in general), and therefore exhibits enormous potential in TE applications.

**Figure 3:**
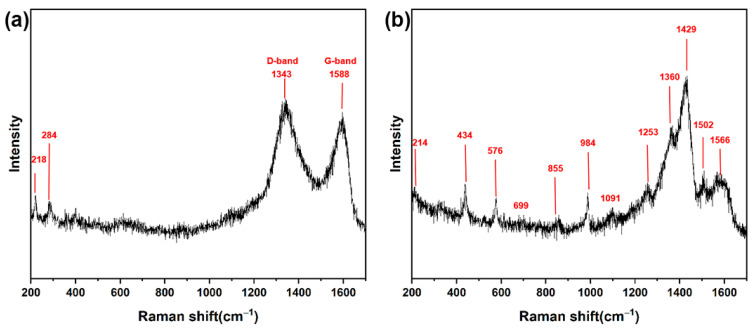
Raman spectra of the obtained hybrid films: (**a**) Fe_3_O_4_@GO/PEDOT:PSS films before acid treatment and (**b**) Fe_3_O_4_@RGO/PEDOT:PSS films after acid treatment.

In order to account for the structural variation in Fe_3_O_4_@RGO/PEDOT:PSS, a Raman spectroscopy experiment was performed. As is depicted in Figure 3a, the pristine Fe_3_O_4_@GO/PEDOT:PSS presents only two main characteristic peaks at 1341 and 1591 cm^−1^, which are associated with the D and G bands of GO, respectively [8,33,35]. Furthermore, two small peaks (centered at 218 and 284 cm^−1^), corresponding to Fe_3_O_4_, also appear in the Raman spectrum [21]. After acid treatment, the most obvious difference is that some novel peaks associated with PEDOT appear. As exhibited in Figure S1, for acid-treated PEDOT:PSS, peaks at 1561 cm^−1^ and 1504 cm^−1^ are assigned to the asymmetric C_α_=C_β_ stretching, while the peak position 1430 cm^−1^ corresponds to the symmetric C_α_=C_β_(–O) stretching in the five-membered ring, 1366 cm^−1^ to the C_β_–C_β_ stretching, 1254 cm^−1^ to the inter-ring C_α_–C_α_ stretching, 1095 cm^−1^ to the C–O–C deformation, 990 cm^−1^ and 576 cm^−1^ to oxyethylene ring deformation, 857 cm^−1^ and 699 cm^−1^ to C–S bonds, and 437 cm^−1^ to SO_2_ bending [36]. With the incorporation of Fe_3_O_4_, a shift of some characteristic peaks is noticeable (see Figure 3b and Figure S1), indicating the interaction between the PEDOT:PSS and Fe_3_O_4_ filler. Furthermore, it is of importance to note that the peak at 1430 cm^−1^ (symmetric C_α_=C_β_(–O) stretching) shifts to 1429 cm^−1^ in the composite sample. In general, the shift of symmetric C_α_=C_β_(–O) stretching vibration is mainly related to the ratio between the benzoid and quinoid conformations. Due to the lack of conjugated π-electrons in C_α_–C_β_, the band red-shift of the symmetric C_α_=C_β_(–O) indicates that more quinoid conformations generate. Namely, with the introduction of Fe_3_O_4_@RGO nanoparticles, there is a conformation transition of the PEDOT molecules from the coiled benzoid to the extended quinoid structure, which facilitates the carrier transformation and is beneficial for electrical performance. Notably, with the acid treatment, the iron oxide characteristic peaks gradually weaken, indicating a decrease in the Fe_3_O_4_ component. This change in Fe_3_O_4_@RGO/PEDOT:PSS would favor the formation of porous nanostructures. When compared with the acid-treated PEDOT:PSS, some PEDOT characteristic peaks become indistinct in the composite film because of the decrease in the PEDOT proportion induced by the addition of the Fe_3_O_4_ filler. All these phenomena indicate the presence of residual Fe_3_O_4_ after acid treatment, and the nanostructures introduced certainly have an influence on the energy performance, which will be further discussed in the subsequent sections.

**Figure 4:**
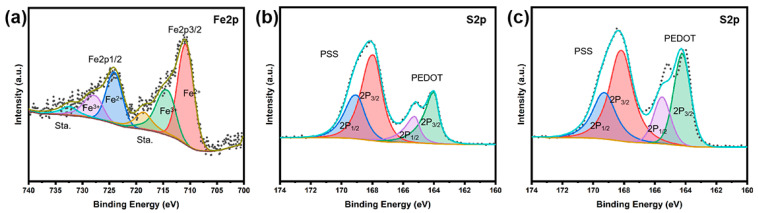
The Fe2p and S2p high-resolution XPS spectra of composite films including fits for the components: (**a**) Fe2p of Fe_3_O_4_@RGO/PEDOT:PSS after acid treatment; (**b**) S2p of Fe_3_O_4_@GO/PEDOT:PSS before acid treatment; (**c**) S2p of Fe_3_O_4_@RGO/PEDOT:PSS after acid treatment.

In order to further investigate the elemental composition and chemical state of the obtained composite films, X-ray electron spectroscopy (XPS) analysis was conducted, and the results are shown in Figure 4 and Figure S2. As depicted in the XPS survey spectrum (see Figure S2), after acid treatment the porous Fe_3_O_4_@RGO/PEDOT:PSS contains elements of Fe, C, O, and S. The high resolution of the Fe2p spectra was decomposed, and the result is shown in Figure 4a. As presented, Fe^2+^ is predominantly correlated with the peaks at 711.2 (Fe2p_3/2_) and 724.5 eV (Fe2p_1/2_), while Fe^3+^ is mainly associated with the peaks at 713.8 (Fe2p_3/2_) and 726.7 eV (Fe2p_1/2_) [37,38]. Additionally, the satellite peaks at 719.1 and 731.8 eV belong to Fe^2+^ and Fe^3+^, respectively [37]. All these phenomena illustrate the presence of residual Fe_3_O_4_ even after a relatively lengthy treatment with HClO_4_ and HI, which serve an important role in electrochemical performance.

To deepen understanding of the influence of acid treatment on structural variations in the composite films, the high-resolution S2p spectra for Fe_3_O_4_@GO/PEDOT:PSS (as prepared) and flexible Fe_3_O_4_@RGO/PEDOT:PSS (after acid treatment) were analyzed, and the results are displayed in Figure 4b,c. As can be seen, the as-prepared Fe_3_O_4_@GO/PEDOT:PSS exhibits two distinct types of S element, because the S2p binding energy of the thiophene unit in PEDOT is very different from that of the sulfonate group in PSS. Namely, there are two types of S; one is related to PSS (high-binding-energy region, 171.8~166.5 eV), and the other is associated with PEDOT (low-binding-energy region, 166.5~162.5 eV). For the S element in PSS, S2p peaks can be divided into S2p_3/2_ and S2p_1/2_ peaks, showing peak positions centered at 168.1 eV and 169.2 eV, respectively. Meanwhile, for the S element in PEDOT, S2p peaks can also be classified into S2p_3/2_ and S2p_1/2_ peaks, centered at 164.1 eV and 165.5 eV, respectively. Comparing Figure 4b with Figure 4c, it can be found that the relative content of PSS to PEDOT decreases distinctly. This ratio decrease can be attributed to conformational variations caused by PSS removal during acid treatment. PSS extraction can be generally quantified by figuring the integral area ratio of the characteristic peaks. The calculation results are shown in Figure S3 (see Supporting Information). A PSS/PEDOT surface element ratio of 2.06 can be achieved for the as-obtained Fe_3_O_4_@GO/PEDOT:PSS composite, whereas the post-treatment using perchloric acid and hydroiodic acid reduces the PSS/PEDOT ratio to 1.48 (for the Fe_3_O_4_@RGO/PEDOT:PSS sample), confirming the PSS removal effect of the acids’ treatment. Because PSS itself is not conductive in nature, its reduction in content can effectively improve the conductive characteristics of the hybrid films, which would not only benefit the enhancement of the electrochemical performance, but also potentially improve the TE properties of the composite electrodes.

### 3.3. Electrochemical Properties of Fe_3_O_4_@RGO/PEDOT:PSS Free-Standing Films

**Figure 5:**
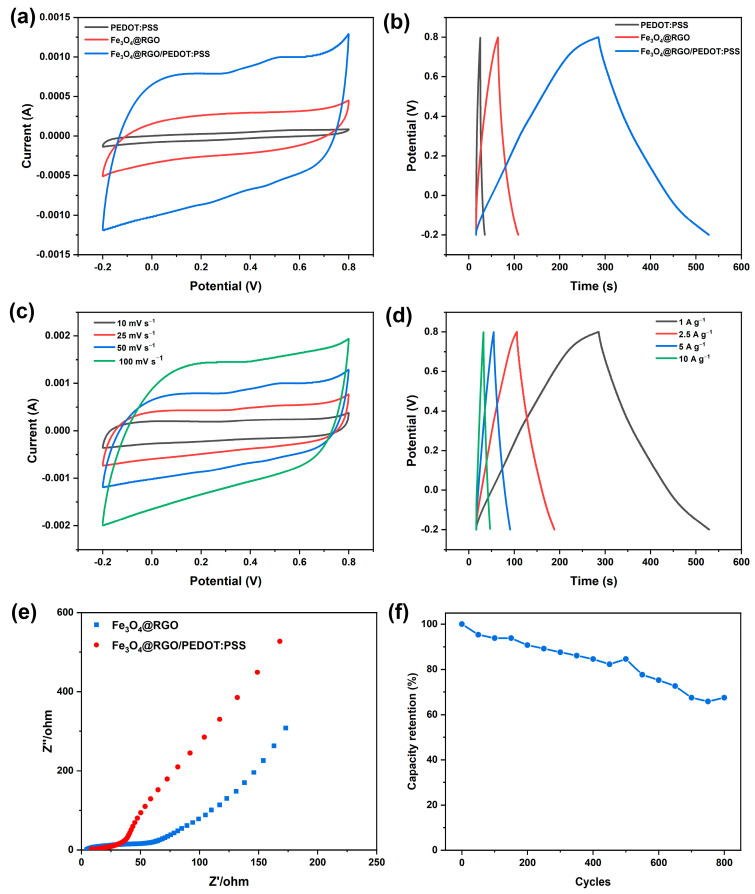
Electrochemical properties of PEDOT:PSS, Fe_3_O_4_@RGO, and Fe_3_O_4_@RGO/PEDOT:PSS electrodes: (**a**) CV curves of samples at a scan rate of 50 mV s^−1^; (**b**) GCD curves of samples at a current density of 1 A g^−1^; (**c**) CV curves of Fe_3_O_4_@RGO/PEDOT:PSS at different scan rates; (**d**) CD curves of Fe_3_O_4_@RGO/PEDOT:PSS at different current densities; (**e**) Nyquist plots of Fe_3_O_4_@RGO and Fe_3_O_4_@RGO/PEDOT:PSS composite samples; and (**f**) cycle stabilities of Fe_3_O_4_@RGO/PEDOT:PSS during the long-term charging/discharging process at a current density of 20 A g^−1^.

CV and GCD were conducted to estimate the electrochemical properties of the electrodes, and the results are presented in Figure 5a,b. It is suggested that the GO skeleton is partially reduced to RGO via HI treatment. Combined with the wrapping effect of the highly conductive PEDOT:PSS, a continuous conductive network is formed, which provides good paths for the transport of ions and rapid redox reactions. This flexible film is expected to be employed as a high-performance film electrode for SCs, working simultaneously as an electrically conducting current collector and active electrode material. As can be observed, the Fe_3_O_4_@RGO/PEDOT:PSS self-supporting film exhibits a quasi-rectangular CV curve at a scan rate of 50 mV s^−1^ (see Figure 5a). For the Fe_3_O_4_@RGO electrode, nearly no redox peak can be seen, owing to its relatively low electric conductivity, whereas for the Fe_3_O_4_@RGO/PEDOT:PSS film, the corresponding redox couples become more clear, which is related to the surface redox reactions between Fe^2+^ and Fe^3+^ [21]. Meanwhile, the area enclosed by the CV curve of the Fe_3_O_4_@RGO/PEDOT:PSS film triples that of Fe_3_O_4_@RGO, representing a larger capacitance. Nevertheless, the pristine PEDOT:PSS itself possesses a smaller CV curve area due to its poor electric conductivity. Promisingly, the nonconductive PSS can be partly removed via acid treatment, which is beneficial for improving conductivity. Meanwhile, Fe_3_O_4_ nanoparticles would be partially etched away via acid treatment, and some macroporous microstructures formed where the reactions take place, benefiting electrolyte transport and the electrochemical redox reactions at the electrolyte/electrode interfaces. The influence of the PEDOT:PSS wrapping effect on the electrochemical performance was further evaluated via GCD measurement, shown in Figure 5b. The specific capacitance of Fe_3_O_4_@RGO is 71.68 F g^−1^, which is comparable with a previous report [10], and can be substantially improved with the introduction of PEDOT:PSS and the subsequent acid treatment. As is shown in Figure 5b, the specific capacitance of Fe_3_O_4_@RGO/PEDOT:PSS can reach a high value of 244.7 F g^−1^ at 1 A g^−1^. Notably, the shape of the Fe_3_O_4_@RGO/PEDOT:PSS curve deviates from the ideal triangular, implying the pseudocapacitive performance is contributed from the Fe_3_O_4_ component. These results are highly consistent with the CV analysis. Therefore, we infer that the introduction of PEDOT:PSS and the subsequent acid treatment contribute to the pseudocapacitance storage of Fe_3_O_4_, resulting in better SC performance.

The effect of the PEDOT:PSS content on the electrochemical performance was also researched, and the results are given in Figure S4. After PEDOT:PSS addition and the following acid treatment, the PEDOT:PSS molecules wrap intimately around the Fe_3_O_4_@RGO framework via strong interactions, generating a continuous and conductive network. Combined with the high electric conductivity of PEDOT:PSS, the porous architecture of Fe_3_O_4_@RGO/PEDOT:PSS facilitates ion transport and fast redox reactions, leading to an enhanced capacitance. Nevertheless, when the addition amount exceeds 28.6 wt% (which becomes 34.06 wt% after acid treatment), the presence of excessive PEDOT:PSS may disrupt the interfacial contact between Fe_3_O_4_@RGO and the electrolyte, causing a significant reduction in redox activity sites. As a result, the capacitive performance of the flexible film is deteriorated. Specifically, when 71.4 wt% (51.08 wt% was left after acid treatment) Fe_3_O_4_@RGO was used, the specific capacity possessed a maximum value. As is exhibited in Figure 5c, further CV curves in a wide range of scan rates (from 10 to 100 mV s^−1^) indicate a good reversibility of the redox reactions. GCD tests of this sample with different current densities were also performed (Figure 5d). They show high specific capacitance of 244.7, 205.0, 181.0, and 146.0 F g^−1^ at 1, 2.5, 5, and 10 A g^−1^, respectively, which is in accordance with the CV curves. From the relative contents of each component (Figure S5), the quantitative contribution of the Fe_3_O_4_@RGO and PEDOT:PSS can be determined. In our previous report, an acid-treated PEDOT:PSS film achieved a specific capacity of around 43.2 F g^−1^ at 1 A g^−1^ [33]. According to this value, the specific capacitance of Fe_3_O_4_@RGO based on its own weight is around 379.0 F g^−1^. These results are comparable or even better when compared with other Fe_3_O_4_-based electrodes [18,19,20,21,22,39,40,41]. The excellent electrochemical performance of the porous Fe_3_O_4_@RGO/PEDOT:PSS could be ascribed mainly to the acid treatment. First, the nonconductive PSS was effectively removed via HClO_4_ and HI immersion. Secondly, for the GO component, some functional groups like oxygen-containing groups would favor rapid ion transfer between the film surface and interior [42]. However, the existence of these functional groups is not beneficial for electron transport, resulting in a low electric conductivity. Via HI treatment, the balance between the oxygen-containing functional groups and conductivity could be properly adjusted for enhancement of the electrochemical properties. Last but not least, the interconnected conductive framework was successfully fabricated via acid treatment (Figure 2e–g), ensuring the fast ion and electron transport.

In addition, EIS measurement was conducted to investigate the ion diffusion and electron transfer resistance of the prepared electrodes. As can be seen from Figure 5e, the Nyquist curve mainly consists of two parts: the high-frequency Nyquist curve is composed of a semicircle, while the low-frequency curve is made up of a nearly straight line. Generally, the semicircle diameter at high frequency is related to the charge transfer resistance (R_ct_). To be specific, the Fe_3_O_4_@RGO possesses a charge transfer resistance of around 50 Ω. It is worthy to note that, after being wrapped with PEDOT:PSS, the electrode shows a marked decline in R_ct_ value. Furthermore, the slope of the Fe_3_O_4_@RGO/PEDOT:PSS electrode at lower frequencies is very similar to that of the Fe_3_O_4_@RGO, indicating that good diffusive behavior of the electrolyte ions is maintained after PEDOT:PSS addition. All results of these EIS analyses are consistent with those collected from the CV and GCD studies, illustrating that the PEDOT:PSS wrapping indeed facilitates efficient ion transport and consequently enhances its capacitance performance. Moreover, a further cyclic stability test was carried out, and the result reveals that even at a very high current density (20 A g^−1^), the Fe_3_O_4_@RGO/PEDOT:PSS integrated electrode film still retains nearly 70% of its original capacitance when it is charged and discharged for 800 cycles (see Figure 5f).

### 3.4. Thermoelectric Performance of Fe_3_O_4_@RGO/PEDOT:PSS Free-Standing Films

**Figure 6:**
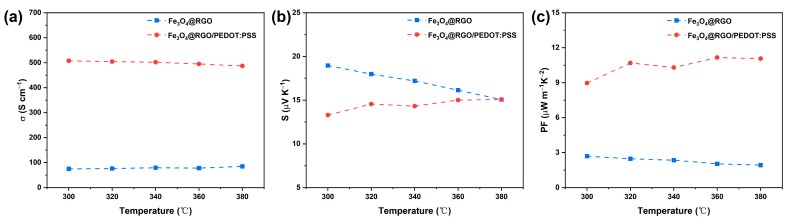
The electrical conductivity (**a**), Seebeck coefficient (**b**), and power factor (**c**) of Fe_3_O_4_@RGO and Fe_3_O_4_@RGO/PEDOT:PSS composite films from 300 to 380 K.

The excellent performance of the hybrid Fe_3_O_4_@RGO/PEDOT:PSS electrodes can be attributed to their unique architecture, which may endow this free-standing film with wider application areas. Herein, we also investigate the thermoelectric properties of this Fe_3_O_4_@RGO/PEDOT:PSS integrated film. For comparison, a Fe_3_O_4_@RGO sample with a similar thickness (Figure S6) was also prepared. Figure 6 shows the variation in the TE parameters of the composite films as a function of absolute temperature. As can be seen, the σ of the Fe_3_O_4_@RGO sample reveals a value of 150 S cm^−1^ and keeps almost constant in the tested temperature interval (Figure 6a). With the incorporation of PEDOT:PSS and the following acid treatment, the obtained Fe_3_O_4_@RGO/PEDOT:PSS integrated film shows a much higher σ value (507.56 S cm^−1^). With increasing temperature, the σ value just shows a slight decrease, and this variation trend is very similar to those reported in the literature [43,44]. Namely, the electric conductivity is not sensitive to the change in temperature. As shown in Figure 6b, all the composite films exhibit positive Seebeck coefficients, illustrating that the predominant charge carriers are holes in the above obtained samples. The Fe_3_O_4_@RGO film shows a Seebeck coefficient of 18.96 μV K^−1^ at room temperature. When the test temperature increases, the S demonstrates a slight downward trend and decreases to 15.07 μV K^−1^ at 380 K. In contrast, the free-standing Fe_3_O_4_@RGO/PEDOT:PSS film possesses an S value of only 13.29 μV K^−1^ at room temperature. Upon increasing the testing temperature from 300 K to 380 K, the Seebeck coefficient presents a visible increasing trend, and achieves an S value of 15.07 μV K^−1^ (the same result with the Fe_3_O_4_@RGO sample) at 380 K. Taking into account the big variation in electric conductivity, the Fe_3_O_4_@RGO/PEDOT:PSS film exhibits a much higher power factor when compared with the Fe_3_O_4_@RGO sample. To be specific (shown in Figure 6c), a maximum PF value of 11.06 μW⋅m^−1^⋅K^−2^ is achieved at 380 K, which is much higher than that of the Fe_3_O_4_@RGO, by nearly four times.

## 4. Conclusions

In summary, we have demonstrated the creation of highly conductive Fe_3_O_4_@RGO/PEDOT:PSS porous films via a facile but efficient method. Benefiting from the supporting effect of the GO framework and the highly conductive networks arising from PEDOT:PSS wrapping and subsequent acid treatment, the ternary Fe_3_O_4_@RGO/PEDOT:PSS composites exhibit excellent electrochemical properties: a high specific capacitance of 244.7 F g^−1^ can be achieved at a current density of 1 A g^−1^; meanwhile, a high level of cycling stability of ~70% is maintained after 800 cycles. The superior electrochemical performance of Fe_3_O_4_@RGO/PEDOT:PSS hybrid films greatly benefits from their unique structures. Notably, the free-standing flexible hybrid films also show relatively high thermoelectric properties, and a high power factor of 11.06 μW⋅m^−1^⋅K^−2^ is reached at 380 K. The novel method proposed in this paper paves an effective way to fabricate and design highly conductive and porous PEDOT:PSS-based composites for electrochemical energy storage and thermoelectric applications.

## Data Availability

All data are contained within the article, and Supplementary Materials are available upon request from the authors.

## Supplementary Materials

The following supporting information can be downloaded at: https://www.mdpi.com/article/10.3390/polym15163453/s1, Figure S1: Raman spectra of the PEDOT:PSS film after acid treatment via HClO_4_; Figure S2: XPS survey spectrum of the porous Fe_3_O_4_@RGO/PEDOT:PSS; Figure S3: PSS/PEDOT ratio calculated for Fe_3_O_4_@GO/PEDOT:PSS samples before acid treatment and Fe_3_O_4_@RGO/PEDOT:PSS after acid treatment.; Figure S4: CV curves of Fe_3_O_4_@RGO/PEDOT:PSS with different PEDOT:PSS content at a scan rate of 50 mV s^−1^: a, the content of PEDOT:PSS is 16.7 wt%; b, the content of PEDOT:PSS is 28.6 wt%; c, the content of PEDOT:PSS is 50.0 wt%; Figure S5: TG curves for Fe_3_O_4_@RGO/PEDOT:PSS flexible film; Figure S6: The thickness of Fe_3_O_4_@RGO and Fe_3_O_4_@RGO/PEDOT:PSS films.
