# Supplementary material for: Fabrication of Highly Conductive Porous Fe3O4@RGO/PEDOT:PSS Composite Films via Acid Post-Treatment and Their Applications as Electrochemical Supercapacitor and Thermoelectric Material
Source: Polymers (Basel). 2023 Aug 18;15(16):3453. doi: 10.3390/polym15163453 (PMC10458617; doi:10.3390/polym15163453)
Supplement: Supplementary file 1 [file polymers-15-03453-s001.zip › polymers-2515461-supplementary.pdf]

polymers

Supporting Information

Fabrication of Highly Conductive Porous Fe_3O_4@RGO/PEDOT:PSS Composite Films via Acid Post-Treatment and Their Applications as Electrochemical Supercapacitor and Thermoelectric Material

Luyao Gao [1,2,3], Fuwei Liu [1,2,3,*], Qinru Wei [1], Zhiwei Cai [1], Jiajia Duan [1], Fuqun Li [1], Huiying Li [1], Ruotong Lv [1], Mengke Wang [1], Jingxian Li [1] and Letian Wang [1]

1 College of Physics and Electronic Engineering, Xinyang Normal University, Xinyang 464000, China
2 Key Laboratory of Advanced Micro/Nano Functional Materials of Henan Province, Xinyang Normal University, Xinyang 464000, China
3 Energy-Saving Building Materials Innovative Collaboration Center of Henan Province, Xinyang Normal University, Xinyang 464000, China
* Correspondence: liufuwei168@163.com

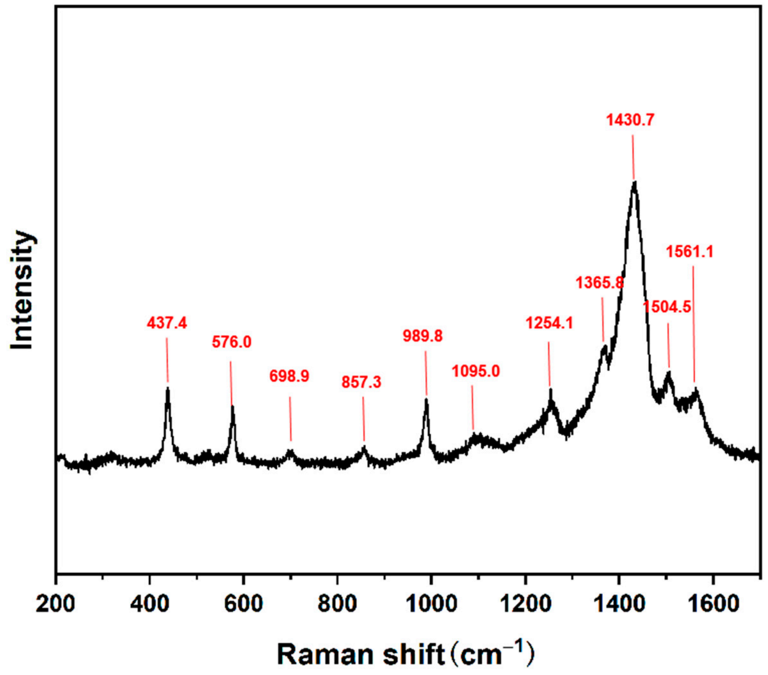

Figure S1. Raman spectra of the PEDOT:PSS film after acid treatment via $HClO_4$.

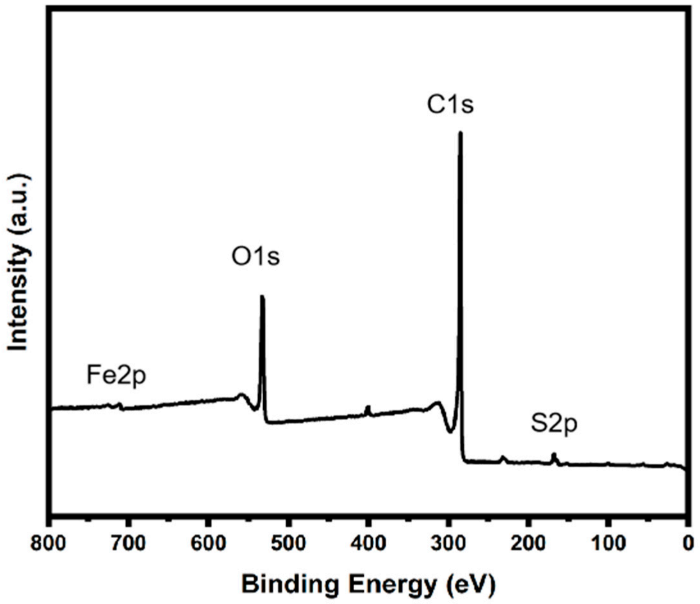

Figure S2. XPS survey spectrum of the porous Fe_3O_4@RGO/PEDOT:PSS.

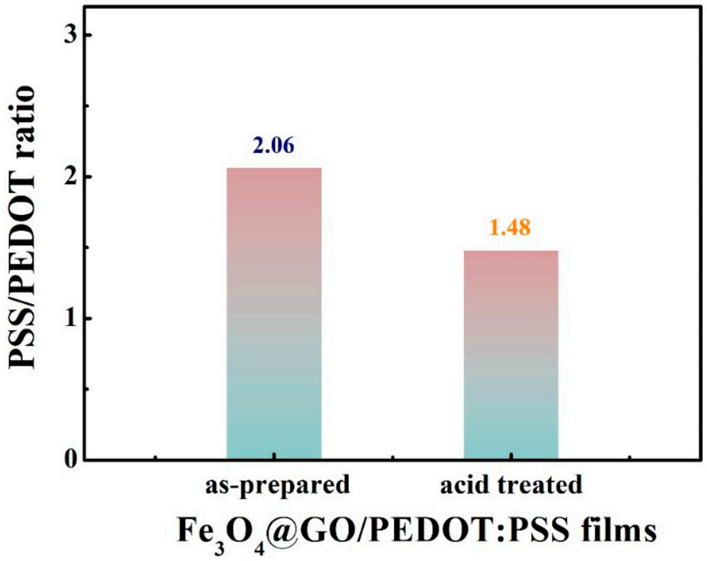

Figure S3. PSS/PEDOT ratio calculated for Fe_3O_4@GO/PEDOT:PSS samples before and Fe_3O_4@RGO/PEDOT:PSS after acid treatment.

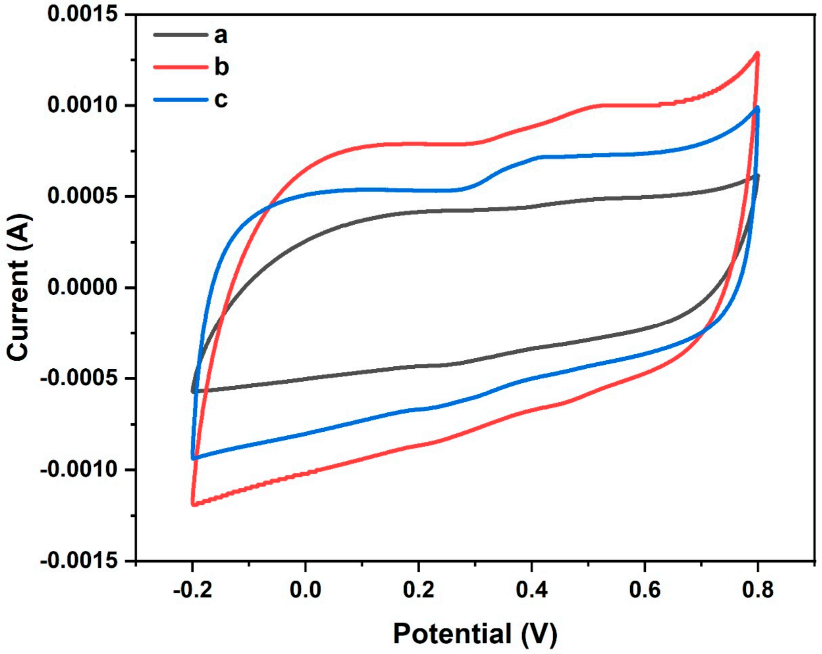

Figure S4. CV curves of Fe_3O_4@RGO/PEDOT:PSS with different PEDOT:PSS content at a scan rate of 50 mV s^{-1}: a, the content of PEDOT:PSS is 16.7 wt%; b, the content of PEDOT:PSS is 28.6 wt%; c, the content of PEDOT:PSS is 50.0 wt%.

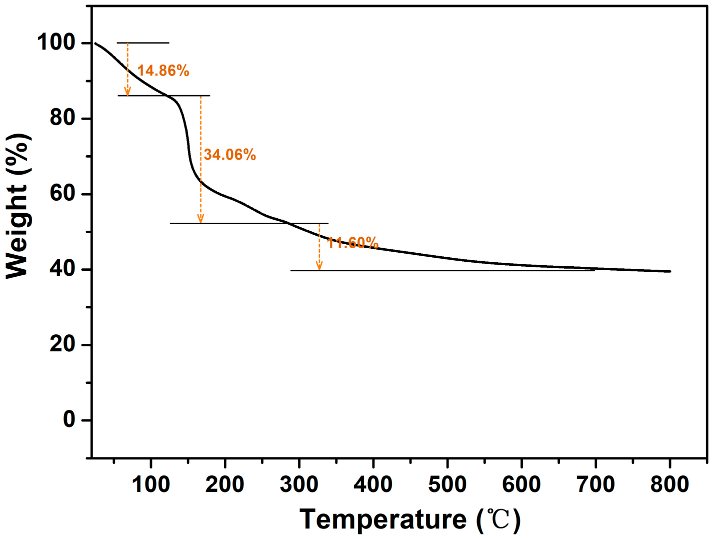

Figure S5. TG curves for Fe_3O_4@RGO/PEDOT:PSS flexible film.

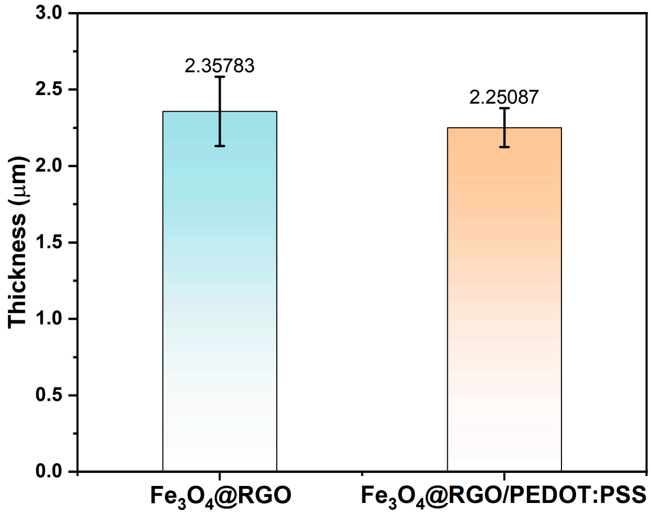

Figure S6. The thickness of Fe_3O_4@RGO and Fe_3O_4@RGO/PEDOT:PSS films.
